# Implementation of a Smartphone application in medical education: a randomised trial (iSTART)

**DOI:** 10.1186/s12909-017-1010-4

**Published:** 2017-09-18

**Authors:** Felipe Martínez, Catalina Tobar, Carla Taramasco

**Affiliations:** 1 Departamento de Salud Pública, Escuela de Medicina, Universidad de Valparaíso, Hontaneda, 2664 Valparaíso, Chile; 2 Área de Investigación y Estudios Clínicos, Clínica Ciudad del Mar, Viña del Mar, Chile; 3 Departamento de Medicina Interna, Hospital Gustavo Fricke, Álvares, 1532 Viña del Mar, Chile; 4 Laboratorio de Información y Tecnología, Escuela de Ingeniería Informática, Universidad de Valparaíso, General Cruz, 222 Valparaíso, Chile

**Keywords:** Medical education, Internal medicine, Smartphones, Student, medical

## Abstract

**Background:**

Smartphones are popular technologies that combine telephone communications and informatics in portable devices. Limited evidence exists regarding their effectiveness in improving academic performance among medical students. This study aims to assess whether a smartphone application could improve academic performance in multiple-choice tests.

**Methods:**

A double-masked randomised trial was held among interns at the School of Medicine of the Universidad de Valparaiso. Participants were randomised to receive an application designed to review key concepts in Internal Medicine and its subspecialties using clinical vignettes. Contents were selected and provided in a format akin to a mandatory national examination required for practising medicine in Chile. Analyses were undertaken under the intention to treat principle and missing data were handled using multiple imputation techniques.

**Results:**

Eighty interns volunteered to participate in this trial, most were female (48 students, 60%) and had a mean age of 25.3 ± 2.2 years. Participants showed significant experience with smartphones, with a median use of 4 years (IQR 3–6 years) and 67 (83.7%) reporting routine use in clinical practice. Intention-to-treat analyses showed significant improvements in performance amongst students allocated to the smartphone application (mean increase of 14.5 ± 8.9 vs 9.4 ± 11.6points, *p* = 0.03). A reduction in total time and mean time per question was also found, which was significant in complete-case analyses (*p* = 0.04).

**Discussion:**

Smartphones were popular among medical trainees. Academic performance was significantly improved by the use of our application, although the overall effect was smaller than expected from previous trials. This study provides evidence that smartphone-based interventions can assist in teaching internal medicine.

**Trial registration:**

ClinicalTrials NCT02723136.

## Background

Smartphones are recent technologies that combine the capabilities of telephone communications and informatics in small portable devices that allow communications and information processing even at the patient’s bedside [1, 2]. As noted in the general public, these devices have shown significant growth in the international medical community [3], niche where they perform functions that range from undergraduate education to health resource management [4, 5]. Several studies have shown that smartphones are frequently used among physicians, medical students and interns, with overall use rates reaching 80%. iOs®-based systems, such as the iPhone®, seem to be the most commonly used platforms [1, 6–8]. The popularity of smartphones is likely to stem from their versatility. Current devices have a wide variety of functions, which can assist in medical decision making, information searches and educational applications, among other uses [8]. Use in clinical practice seems to be more common among women, people with an interest in new technologies and those with prior experiences with these platforms [7].

Despite this popularity, there is limited evidence regarding the effectiveness of smartphone use in improving academic performance among medical students [9]. While there is a wide availability of applications and resources available for these platforms, only a few randomised trials have addressed their effectiveness in improving academic performance. In 2011, Low and coworkers published one of these studies using objective clinical competence scores as a primary endpoint [10]. The latter trial reported a statistically significant improvement of roughly 15% in the academic performance of students allocated to receive the application. Similar findings were seen in a second, before & after, study that was conducted among Obstetrics & Gynecology residents [11].

Since 2003, a national examination for undergraduate medical students that have completed their internships is carried out in Chile. This exam (Examen Unico Nacional de Conocimientos en Medicina - EUNACOM) is designed to assess the overall knowledge and practical skills that any medical student should attain before practising medicine in the country. Its confection and administration are regulated by law, and its oversight has been delegated to the Association of Faculties of Medicine of Chile (ASOFAMECH). EUNACOM is made of two sections, theoretical and practical, and is considered qualifying to practise medicine in Chile. The contents of both sections are of public knowledge and include 1543 items distributed according to the curricular time spent training in different areas of medicine, with special emphasis on internal medicine and its subspecialties [12]. The theoretical component is evaluated using 180 multiple-choice questions delivered in two 90-min sessions. Additionally, EUNACOM provides professional title validation or equivalencies for foreign physicians who wish to practice medicine in Chile. Given the importance of this exam, several medical schools have implemented preparation courses for their students. However, the methodologies used in the latter courses are heterogeneous, and uncertainty exists regarding the best way in which contents should be delivered.

This study aims to determine whether the implementation of a smartphone application designed to assist in delivering key concepts relevant to internal medicine might improve academic performance in EUNACOM.

## Methods

**Fig. 1:**
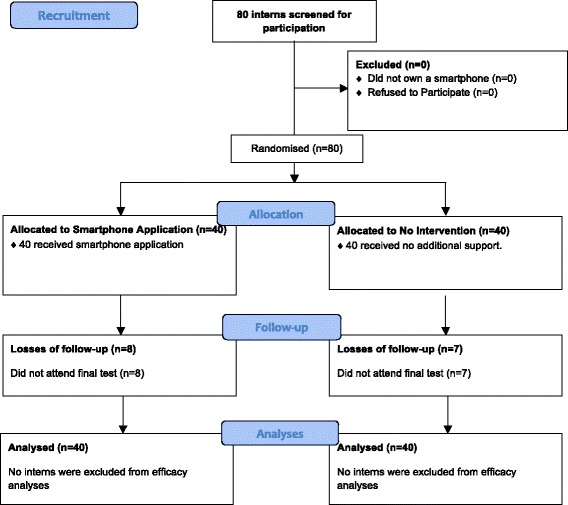
CONSORT Study Flowchart. This figure depicts participant’s flow within the *iSTART* study

iSTART is a double-masked randomised trial that was held among medical students at the School of Medicine of the Universidad de Valparaíso, Chile. The study protocol has been drafted in compliance with the Consolidated Standards of Reporting Trials (CONSORT) statement as in its version adapted for trials evaluating non-pharmacological interventions [13, 14]. The complete protocol was registered in March 2016 at *clinicaltrials.gov* (NCT02723136) and can be reviewed at https://clinicaltrials.gov/ct2/show/NCT02723136?term=NCT02723136&rank=1. A flowchart describing participant recruitment and overall study design is shown in Fig. 1.

### Participants

Eligible participants were medical interns coursing their last year of training at the School of Medicine of the Universidad de Valparaiso, who had a personal Smartphone with an iOs®- or Android®-based operating system. Only those that did not wish to participate were excluded from this study. Informed consent was obtained from all participants.

Every student sat a baseline 90-question test aimed to resemble EUNACOM (see below) and were randomised to receive a smartphone-based application training afterwards. Randomisation was carried out using permuted blocks by a statistician that was unaware of treatment allocation. Allocation sequences were concealed from other researchers participating in this study. All participants were asked to complete an entry form with basic demographic data, including age, sex, year of training and prior experiences with smartphones or similar platforms (i.e. tablets). Data regarding academic performance was obtained from the University, including qualifications relevant to the area of Internal Medicine.

### Interventions

Students allocated to receive the active intervention received a downloadable application that was installed in their smartphones. Those allocated to the control group did not receive any additional training for EUNACOM. The mobile application was devised by a team of informatic engineers and physicians and made available for free at the App Store® and PlayStore® for both iOs® and Android® operating systems. In order to monitor adherence, the application required an active internet connection for operation. Students also received a brief (5-min) description on functionality that was also made available in text form as a part of the software.

Contents were primarily directed at the area of Internal Medicine, which is the most important specialty within EUNACOM. It included a series of questions in the form of brief clinical vignettes constructed in a format similar to the one described in EUNACOM’s website [15]. In short, these vignettes correspond to clinical scenarios against which the student must answer a key aspect relevant to the diagnosis, management or monitoring of several diseases. These multiple-choice questions must be answered from five possible options, with only one being the correct answer. The depth of knowledge required to answer was established using the provisions of the EUNACOM agenda [12]. All contents of the application were designed by two internists with 5 years experience in developing questions for the exam. Examples of these vignettes are provided in the Additional file 1.

The application had two modes to provide the aforementioned inquiries. In the first, study mode, students were not given time constraints to answer the clinical vignettes. Whenever an answer was provided, instant feedback was delivered alongside a brief explanation of the key concept that was being assessed by the inquiry. In the second, training mode, participants had a restricted time window to provide answers. This mode was designed because of a perceived difficulty amongst interns in managing time in answering questions in previous simulations of the exam. A default of 60 s was established, but the application allowed the user to modify this timeframe to 30 or 90 s. Students had knowledge regarding their individual performance in both modes, but no additional feedback in terms of concept review was provided in training mode.

### Outcomes

The primary outcome is the mean change in overall scores in a 90-question practise test designed to resemble EUNACOM between groups. The final test did not repeat any of the questions used within the application that was delivered to students and was held 4 weeks after randomisation. This timeframe was selected in order to allow students to practise and study internal medicine with the application given the extent of contents required by EUNACOM. Simulation tests were used because of the impossibility to use the actual exam as part of this study, since it is managed independently from universities and kept in strict reserve by ASOFAMECH. However, previous data has shown that both simulation exams (baseline and final) have good correlation with overall EUNACOM scores (*r* > 0.7, *p* < 0.001), as well as an excellent diagnostic accuracy for detecting students at risk of failing the exam(area under the ROC curve 0.95, 95% CI 0.90 to 0.99) and identifying students that will obtain high scores in the review (AUC 0.80, 95% CI 0.71 to 0.88, unpublished data). The correction of both practice tests was undertaken by reseachers that were kept unaware of allocation.

A secondary endpoint was to establish differences in the average time required to answer clinical vignettes. In order to allow reliable comparisons to be made, exams were conducted electronically and under supervision by the research team, thus allowing an objective assessment of the total time required to complete the review. Data regarding adherence was also collected.

### Statistical analyses

#### Sample size

Sample size was calculated using data regarding overall perfomance in prior experiences with practise exams and estimates from a randomised trial [10]. It was calculated that a sample size of 64 participants (32 per group) would be required to obtain 80% power to detect an absolute difference of 5 points between groups, assuming a standard deviation of 7 points for both groups at standard significance levels (two-tailed α of 5%). In order to correct for up to 20% losses of follow-up, it was sought to randomise 75 participants. All estimates were calculated using nQuery Advisor® 3.0 for windows.

#### Analysis plan

Basic descriptive statistics (means, medians, proportions, interquartile ranges -IQR-, etc) were performed to assess the characteristics of the study sample. Fisher’s exact test was used to evaluate univariate association of categorical variables. Quantitative variables were compared using Mann-Whitney or Student’s T tests according to data distribution and variances. Ninety-five percent confidence intervals were constructed whenever appropriate. Missing data relevant to the primary and secondary outcomes were handled using multiple imputation techniques. In order to reduce sampling variability due to the imputation process, 20 datasets were generated for every variable with missing data. Predictor variables were included in this procedure using linear regression for data showing normal distributions. Predictive mean matchings were preferred to impute data for variables with skewed distributions. All analyses were undertaken by a statistician who was unaware of participant allocation using Stata v12.0® (StataCorp LP, 1996–2016) under the intention-to-treat principle, but complementary complete-case analyses were conducted as part of multiple imputation techniques.

## Results

### Participant characteristics

A total of 80 interns were eligible for this study, and all volunteered to participate. Most were female (48 students, 60%) with a mean age of 25.3 ± 2.2 years and had spent a median of 6 years in medical school (IQR 6–7 years). Eighteen (22.5%) had repeated at least one course, and the median number of repetitions was 1 (IQR 1–3). The median time using smartphones was of 4 years (IQR 3–6 years). Most interns reported routine use of smartphone applications in daily practice (67 students, 83.7%), but only a third of them acknowledged using them for academic purposes (31 students, 38.8%). The most common operating system was Android® (51 students, 63.8%). No relevant imbalances in study groups were seen at baseline. A detailed description of these contrasts and additional information regarding study participants is provided in Table 1.

**Table 1:** Baseline Participant Characteristics

Characteristic	Smartphone Application (*n* = 40)	No Intervention (*n* = 40)	Total	*P*-Value
General and Academic characteristics				
Mean Age (years) (SD)	25.6 ± 2.7	24.9 ± 1.5	25.3 ± 2.2	0.18^1^
Female sex (n, %)	27 (67.5%)	21 (52.5%)	48 (60%)	0.25^2^
Median time in medical school (years) (IQR)	6 (6–7)	6 (6–7)	6 (6–7)	0.35^1^
Campus Valparaiso (n, %)	28 (70%)	28 (70%)	56 (70%)	1^2^
Course repetition (n, %)	10 (25%)	8 (20%)	18 (22.5%)	0.79^1^
Median number repetitions (IQR)	1.5 (1–3)	1 (1–3)	1 (1–3)	0.26^3^
Internal Medicine Internship Grade (SD)	6.3 ± 0.4	6.3 ± 0.4	6.3 ± 0.4	0.84^1^
Internal Medicine Undergraduate Examination Grade (SD)	5.4 ± 0.7	5.4 ± 0.7	5.4 ± 0.8	0.91^1^
Experience with Smartphones				
Median time using smartphones (years) (IQR)	4 (3–5)	4 (3–6)	4 (3–6)	0.89^1^
Smartphone use in clinical practice (n, %)	34 (85%)	33 (82.5%)	67 (83.7%)	1^2^
Smartphone use for academic purposes (n, %)	16 (40%)	15 (37.5%)	31 (38.8%)	1^2^
Operating system (n, %)				
Android®	25 (62.5%)	26 (65%)	51 (63.8%)	1^2^
iOs®	15 (37.5%)	14 (35%)	29 (36.2%)	
Performance in Baseline Test				
Mean overall score (SD)	40.3 ± 11.0	41.8 ± 11.2	41.1 ± 11.1	0.53^1^
Mean total time (minutes) (SD)	65.2 ± 26.3	66.0 ± 28.0	65.6 ± 27.0	0.89^1^
Mean time per question (seconds) (SD)	44.3 ± 18.6	45.5 ± 21.7	44.9 ± 20.1	0.80^1^

^1^Student’s T Test. ^2^Fisher’s Exact Test^3^Mann-Whitney U TestSD: Standard Deviation. IQR: Interquartile range

### Intervention effects

The mean score in the baseline test was of 41.1 ± 11.1 points, and mean total time needed for completion of the latter review was 65.6 ± 27.0 min. Scores and completion times were similar between groups at baseline. Sixty-five interns (81.3%) sat the final test 4 weeks after randomisation. In both groups, a significant increase in overall scores was seen, which tended to be greater among interns allocated to receive the smartphone application. Participants allocated to no intervention showed an increase of 10.6 ± 11.7 points (*p* < 0.001) from baseline, while interns who received the smartphone application improved their scores by 16.2 ± 8.3 points (*p* < 0.001).

**Fig. 2:**
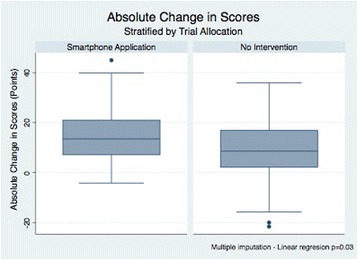
Absolute change in scores between study groups. These boxplots compare the observed differences in perfomance in two simulation tests aimed to resemble EUNACOM

Intention to treat analyses using multiple imputation techniques showed significant differences between study groups. Missing scores were imputed using results from the baseline test and allocation as independent variables in linear regression analyses. On average, interns allocated to the smartphone application had an increase in scores that was 5 points (9%) higher than those observed in the no-intervention group (*p* = 0.03). Similar trends were seen when complete-case analyses were undertaken. When overall scores were analysed, an absolute difference of 3.5 points was observed between groups in favour of those allocated to the smartphone application, but statistical significance was not reached (*p* = 0.22). Study outcomes are briefly summarised in Table 2 and Fig. 2.

**Table 2:** Study Outcomes

Outcome	Smartphone Application	No Intervention	Mean Difference	*P*-Value
Intention to Treat Analyses (Multiple Imputation)				
Mean overall score (points) (SD)	56.1 ± 14.5	52.2 ± 10.3	3.5	0.22^1^
Absolute change in overall score (points) (SD)	14.5 ± 8.9	9.4 ± 11.6	5.0	0.03^1^
Mean total time (minutes) (SD)	62.2 ± 20.4	70.8 ± 21.3	8.5	0.08^2^
Mean time per question (seconds) (SD)	41.2 ± 14.5	46.9 ± 13.6	5.7	0.08^2^
Complete-Case Analyses				
Mean overall score (points) (SD)	56.1 ± 12.9	52.2 ± 9.4	3.9	0.17^2^
Absolute change in overall score (points) (SD)	14.6 ± 7.4	9.6 ± 10.5	5.0	0.03^2^
Mean total time (minutes) (SD)	61.5 ± 19.7	71.5 ± 18.7	10.0	0.04^2^
Mean time per question (seconds) (SD)	41.0 ± 13.2	47.7 ± 12.5	6.7	0.04^2^

*SD* Standard Deviation

^1^Estimates obtained by pooling results across 20 multiply imputed data sets

^2^Student’s T Test

**Fig. 3:**
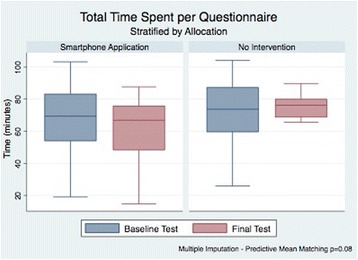
Total time spent per questionnaire. These boxplots show the changes in time required to complete the simulation tests used within iSTART between study groups

Students allocated to the smartphone application showed reductions in the total time needed to complete the final examination and the mean time spent per question. Intention-to-treat analyses showed a nonsignificant reduction of 8.5 min for the first outcome and 5.7 s for the latter (*p* = 0.08 for both). This estimate was calculated using predictive mean matching due to the skewed nature of time data, using allocation and both baseline performance and time required to complete the first examination as predictor variables. These differences were more conservative than the ones observed in complete-case analyses. Among participants who attended the second assessment, a 10-min reduction in overall time and a 6.7 s reduction in mean time per question were found, and both reached statistical significance (*p* = 0.04). Total times spent by participants answering both baseline and final questionnaires are shown in Fig. 3.

### Adherence

The most popular mode amongst participants was study mode, which was used by 34 participants allocated to the intervention (85%, 95% CI 70.2–94.2%). The median number of questions answered during the 4-week intervention period was 258 (IQR 66–415), and the median number of completed questionnaires per participant was 15 (IQR 14–21). Participants used the application’s training mode less frequently, with only 12 students (30%, 95%CI 16.6–46.5%) registering any activity during this trial. The median number of tests answered by these students was 2 (IQR 1–4), which translated in 90 (IQR 45–180) time-limited questions (Table 3).

**Table 3:** iSTART adherence rates

Endpoint	Smartphone Application	95% Confidence Interval
Study Mode		
Proportion of students using this mode (n, %)	34 (85%)	70.2–94.2%
Median number of questions answered (IQR)	258 (66–415)	96–376
Median number of questionnaires completed (IQR)	15 (14–21)	13–22
Training Mode		
Proportion of students using this mode (n, %)	12 (30%)	16.6–46.5%
Median number of questionnaires answered (IQR)	2 (1–4)	1–5

*IQR* Interquartile range

## Discussion

Smartphones are commonly used devices among medical trainees. In this study, every eligible student had at least one of these gadgets at their disposal, and most reported considerable experience using them in their everyday lives. This popularity makes these platforms attractive targets to design and develop interventions for medical training. However, only a handful of randomised trials that address smartphone applications with educational purposes are available in the literature. Most of them have been conducted among postgraduate students undergoing specialty training, and used smartphones as cognitive aids concomitant to assessments of very specific competences [10, 16, 17].

We found that the provision of key concepts in internal medicine using smartphones was a feasible option that also translated in significant improvements in academic performance among medical interns. The observed progress was significant even for a relatively brief intervention that was also self-administered by our students, which adds to the relevance of our findings. Our results are in concordance with the ones observed in similar experiences. In 2011, Low and coworkers [10] published a randomised trial assessing iResus®, an iOs®-based application aimed at improving performance of an advanced life support provider in an emulated medical emergency. The application was designed to provide a quick reference to algorithms and drug dosages to assist in the management of resuscitation efforts. Thirty-one physicians who had already completed an advanced life support course within the previous 4 years were randomised to receive iResus® as a cognitive aid or no additional support during a simulated cardiac emergency. Performance was measured using a validated scoring system. Participants allocated to iResus® showed median scores that were 12.5 (14%) points higher than those seem among students without any further cognitive aids (*p* = 0.02). Similar findings were seen in a larger study by Hand an coworkers [16], in which 111 residents were randomised to a smartphone-based decision support tool aimed at improving adherence to the American Heart Association Guidelines on Perioperative Cardiac Evaluation. Use of the decision support tool resulted in a 25% improvement in adherence to guidelines (*p* < 0.001), and participants made 77% fewer incorrect responses in two standardised tests.

Although our findings are similar to the ones seen in the aforementioned trials, our estimates are far more conservative than the ones observed by Low [10] and Hand [16]. This might be explained by the fact that our intervention was not devised to be used concomitantly to assessments as a cognitive aid, but rather as a complementary resource to facilitate study of internal medicine as a discipline. In addition, it should be considered that the scope of contents established as key by the designers of EUNACOM is broader than the ones required by guidelines aimed at aiding clinicians in the management of specific healthcare issues; thus resulting in an apparent reduction of the intervention’s benefits.

Intention-to-treat analyses also showed a nonsignificant trend towards a reduction in total test times and mean time spent per inquiry. A post-hoc power calculation showed that the estimated power for this contrast was of only 45%, thus making insufficient power a reasonable possibility to explain this observed lack of statistical significance. Nonetheless, the observed reduction of 8.5 min is relevant for interns planning to undertake EUNACOM, and is likely to be the result of practice in answering multiple-choice questions. Clinical vignettes are constructed using certain features that are typical of certain conditions, thus leading to patterns that students exposed to the application might have been able to recognise faster than those allocated to the no-intervention group. It could also be argued that students allocated to the intervention also had more experience answering questions on an electronic platform, thus resulting in familiarity with the interface that might have explained these findings. However, this explanation seems rather unlikely considering the vast experience with smartphone applications that participants had in this study.

Given that the intervention was devised to be self-administered by students, adherence was a key aspect to assess while conducting our study. Thirty-four out of 40 participants (85%) used the application’s study mode to review internal medicine in this trial, which was very satisfactory. Furthermore, the median number of questions and questionnaires completed was more than adequate considering the relatively brief timeframe in which this study was conducted. Only a minority of students allocated to the intervention (12 students, 30%) used the applications’ training mode, the sole feature within the application in which a time restraint to respond clinical vignettes was applied. This obvious contrast in use rates reached statistical significance (*p* < 0.001), and might be explained by performance pressure. It is possible that interns felt discouraged to undertake activities that recorded results in a manner similar than the one used in the actual EUNACOM. Participants could have associated underperforming in these exercises with a potential for poor results in the exam, thus leading to the observed use rates. Feedback provided by this mode did not include a revision of the key concept in internal medicine that was being assessed, thus possibly making pressure for delivering high scores more tangible. Furthermore, interns were warned that time-limited exercises were accessible only once during our trial, which might have led to lesser use rates in order to save this component of the application after the reviewable contents (study mode) were completed. Given these explanations and the fact that EUNACOM applies a time limit of 60 s per question, future interventions aimed at improving performance in this and/or similar tests should not disregard applying time restraints as part of their strategies. Exploring motivations to use these types of applications should be considered in future qualitative research.

### Strengths and limitations

Our study is strengthened by randomisation, which greatly helps controlling biases due to selection and confounding. Contents within the application were designed by internists with experience in developing questions that resemble those used in EUNACOM. Previous data available at our centre had shown good correlations with overall scores and those specific with internal medicine within the review, which has translated in excellent diagnostic accuracy in detecting students at risk of failing the examination. We also conducted active monitoring of the application’s use, which greatly helps understanding our results and represents a key element when evaluating interventions that are self-delivered by students. These data are likely to be helpful for the design of future versions or similar applications.

Several limitations need to be taken into consideration when analysing our results. The first is that a significant proportion of students did not attend the final examination (18.7%), which resulted in the loss of key information regarding study outcomes. We chose to mitigate this event by using multiple imputation techniques, which have been established as one of the best methods available to handle missing data in randomised trials [18, 19]. Uncertainty always exists when estimates from multiple imputation are used to allow the conduction of intention-to-treat analyses. This stems from the fact that the “missing completely at random” assumption of missing data is hard to confirm in practice [18]. We did not find any contrasts between participants who completed our study and those who did not, and estimates from complete case analyses were very similar to the ones obtained from multiple imputation. Both facts bring reassurance regarding the reliability of our imputed values. Another limitation stems from the impossibility to mask participants to the intervention, which could have resulted in the application’s contents being shared across study groups. This would result in a minimisation of the intervention’s effects between groups, and thus might explain the smaller-than-expected difference that was found in this trial. Costs are always a relevant concern when implementing interventions in medical education. In this case, an investment of 50.000USD was required to develop the application and its key contents, which was covered entirely by the research team. Most expenses were incurred in human resource honoraria. Although this might be seen as a significant barrier to implementation, it should be considered that after this initial investment, the application was inexpensive to maintain, only requiring monthly payments for a server and a part-time engineer to oversee its functionality and data collection processes. Development costs can be mitigated by working with volunteers or in collaboration with other institutions or academic departments which might use this application as a platform for additional contents. The modular design of our application allows unrestricted upload of questionnaires that are not limited to undergraduate training, thus opening a potential for postgraduate and continuous medical education. In addition, institutions interested in implementing these kind of applications might consider allowing access to individuals by paying download/subscription fees in order to ensure sustainability over time.

## Conclusion

In summary, this randomised trial showed that the provision of a smartphone application designed to emulate EUNACOM was successfully implemented amongst medical interns. Its use was associated with significant improvements in academic performance, and is likely to be chiefly explained by the direct provision of concepts in Internal Medicine in form of clinical vignettes. The observed high adherence rates amongst interns make these kinds of interventions a promising field to develop in the future of medical education.

## Additional file

Additional file 1: Examples to Clinical Vignettes. (DOCX 112 kb)

## Abbreviations

ASOFAMECH: Asociación de Facultades de Medicina de Chile
CI: Confidence Interval
EUNACOM: Examen Unico Nacional de Conocimientos en Medicina
IQR: Interquartile Range
iSTART: Implementation of a Smartphone Application in Medical Education: A Randomised Trial
