# Supplementary material for: Implementation of a Smartphone application in medical education: a randomised trial (iSTART)
Source: BMC Med Educ. 2017 Sep 18;17:168. doi: 10.1186/s12909-017-1010-4 (PMC5604333; doi:10.1186/s12909-017-1010-4)
Supplement: Additional file 1: — Examples to Clinical Vignettes. (DOCX 112 kb) [file 12909_2017_1010_MOESM1_ESM.docx]

**Supplementary Appendix**

**Examples to Clinical Vignettes**

1. A 30 year-old male with a history of HIV infection presents to the emergency department due to shortness of breath and fever (38ºC). Due to desaturation, he is placed under supplementary oxygen (FiO2 35%). A chest x-ray reveals infiltrates in the right lower lobe.

**¿Which is the most likely agent causing the infection?**

a) Staphylococcus aureus

b) Streptococcus pneumoniae

c) Pneumocystis jiroveci

d) Mycoplasma pneumoniae

e) Haemophilus influenzae

**Answer:** **B.** The most common agent causing community-acquired pneumonia in Chile is *Streptococcus pneumoniae*. This includes patients living with HIV.

2. A 65 year-old female with a history of a myocardial infarction comes to the emergency room because of palpitations and shortness of breath. Her pulse is 150 bpm, and an EKG shows a regular tachycardia with a QRS width of 100msec.

**¿What is the most likely diagnosis?**

a) Sinus tachycardia

b) Ventricular tachycardia

c) Paroxysmal supraventricular tachycardia

d) Atrial fibrillation

e) Atrial flutter

**Answer:** **E.** A regular, narrow-complex tachycardia in a patient with a coronary history is most likely caused by an atrial flutter. The 150bpm ventricular rate is very suggestive of this diagnosis. Ventricular tachycardias present with wide (>120msec) QRS intervals.

3. A 55 year-old diabetic female is brought to the hospital due to fever and shortness of breath. On examination, she's tachypneic, tachycardic and has dry mucous membranes. Laboratory analyses showed a blood glucose of 480mg/dL, ketone bodies, HCO3^-^ 14mEq/Lt, Na^+^ 150mEq/Lt and K^+^ 5.0mEq/Lt.

**¿Which treatment should be started next?**

a) Insulin in a continous infusion

b) Insulin bolus followed by a continuous infusion

c) Isotonic (0.9%) saline

d) Sodium bicarbonate

e) Potassium chloride

**Answer:** **C**. The first step in managing patients with diabetic ketoacidosis is to restore tissue perfusion by providing isotonic fluids.
